# Furin cleavage of the SARS-CoV-2 spike is modulated by *O*-glycosylation

**DOI:** 10.1073/pnas.2109905118

**Published:** 2021-11-03

**Authors:** Liping Zhang, Matthew Mann, Zulfeqhar A. Syed, Hayley M. Reynolds, E. Tian, Nadine L. Samara, Darryl C. Zeldin, Lawrence A. Tabak, Kelly G. Ten Hagen

**Affiliations:** ^a^Developmental Glycobiology Section, National Institute of Dental and Craniofacial Research (NIDCR), NIH, Bethesda, MD 20892-4370;; ^b^Section on Biological Chemistry, NIDCR, NIH, Bethesda, MD 20892-4370;; ^c^Structural Biochemistry Unit, NIDCR, NIH, Bethesda, MD 20892-4370;; ^d^Division of Intramural Research, National Institute of Environmental Health Sciences, NIH, Research Triangle Park, NC 27514

**Keywords:** SARS-CoV-2, *O*-, glycosylation, furin, spike, COVID-19

## Abstract

The SARS-CoV-2 coronavirus responsible for the global pandemic contains a novel furin cleavage site in the spike protein (S) that increases viral infectivity and syncytia formation in cells. Here, we show that *O*-glycosylation near the furin cleavage site is mediated by members of the GALNT enzyme family, resulting in decreased furin cleavage and decreased syncytia formation. Moreover, we show that *O*-glycosylation is dependent on the novel proline at position 681 (P681). Mutations of P681 seen in the highly transmissible alpha and delta variants abrogate *O*-glycosylation, increase furin cleavage, and increase syncytia formation. Finally, we show that GALNT family members capable of glycosylating S are expressed in human respiratory cells that are targets for SARS-CoV-2 infection. Our results suggest that host *O*-glycosylation may influence viral infectivity/tropism by modulating furin cleavage of S and provide mechanistic insight into the role of the P681 mutations found in the highly transmissible alpha and delta variants.

## Significance

The novel SARS-CoV-2 coronavirus that is responsible for the global pandemic contains a unique insertion of four amino acids within the spike protein (S). Furin cleavage at this novel insertion site has been shown to increase pseudoviral infectivity and syncytia formation. Here we show that *O*-glycosylation by certain GALNT family members decreases furin cleavage of S and decreases syncytia formation. Moreover, we show that P681 mutations found in the highly transmissible alpha and delta variants decrease *O*-glycosylation, which increases furin cleavage and syncytia formation. Our results highlight how host-mediated *O*-glycosylation may influence viral infectivity and how mutations in the recent alpha and delta variants may circumvent this.

**Fig. 1.**
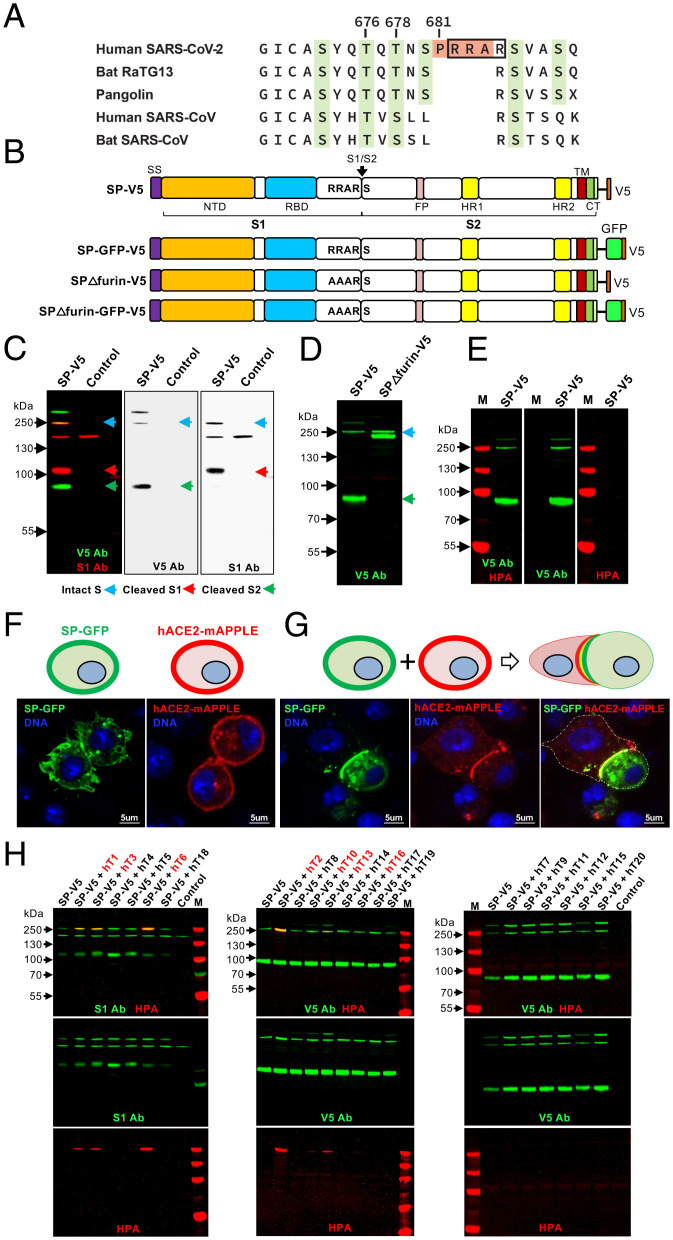
Specific GALNTs *O*-glycosylate the SARS-CoV-2 S protein. (*A*) Sequence alignment of the S1/S2 junction region of the S protein from related betacoronaviruses, highlighting the PRRA insertion unique to SARS-CoV-2 (in orange) and potential sites of *O*-glycosylation (in green). The furin cleavage site is boxed. Numbering corresponds to the human SARS-CoV-2 sequence. (*B*) Diagram of full-length WT SARS-CoV-2 S protein (SP) or version with the furin site mutated (RRAR to AAAR; SPΔfurin) linked to either V5 or GFP-V5. The site of furin cleavage at the S1/S2 junction is shown with an arrow. NTD, N-terminal domain; RBD, receptor-binding domain; FP, fusion peptide; HR1, heptad repeat 1; HR2, heptad repeat 2; TM, transmembrane domain; CT, cytoplasmic tail. (*C*) Expression of SP-V5 in *Drosophila* S2R^+^ cells results in full-length S (blue arrow), which can be detected by either the V5 antibody (V5 Ab; green) or the S1 antibody (S1 Ab; red). Also detected in these cells are the S1 fragment (detected by the S1 Ab; red arrow) and the S2 fragment (detected by the V5 Ab; green arrow) that result from cleavage of S. The band running at >250 kDa likely represents trimers/multimers of S2, as it is only detected by the V5 Ab. Control lane is from cells transfected with the empty vector. (*D*) Mutation of the furin cleavage site (SPΔfurin-V5) results in the presence of only full-length S (detected by V5 Ab; blue arrow). Doublet seen in the SPΔfurin-V5 lane represents full-length S with and without the signal sequence. (*E*) No evidence of *O*-glycosylation (via HPA staining; red) is seen for S (detected by V5 Ab; green) in this cell background. (*F*) *Drosophila* S2R^+^ cells were transfected with either SP-GFP-V5 (green) or the human ACE2 receptor (hACE2-mAPPLE, red) to demonstrate cell surface expression. (*G*) Cells expressing either SP-GFP-V5 or hACE2 were then mixed as shown in the diagram and imaged to reveal that S expressed in these cells maintains the ability to bind to the hACE2 receptor. Nuclear staining is shown in blue. (*H*) SP-V5 was cotransfected with each member of the human *GALNT* family (hT1-20) and *O*-glycosylation was assessed by staining with the *O*-glycan–specific lectin HPA (red). S was detected via staining with the S1 Ab (green) or V5 Ab (green). GALNT family members that glycosylate S are shown in red at the top of the panel. Control lanes are from cells transfected with the empty vector. M, markers. Marker size is shown in kilodaltons on the side of each panel.

The novel severe acute respiratory syndrome coronavirus-2 (SARS-CoV-2), which is responsible for the current coronavirus disease 2019 (COVID-19) pandemic, contains a unique insertion of four amino acids (PRRA) at the S1/S2 boundary of the spike protein (S) that is not present in SARS-CoV and other related coronaviruses (1) (Fig. 1*A*). The S protein present in the viral envelope is responsible for binding to host cells and mediating viral entry into cells after S undergoes a number of cleavages. Interestingly, this PRRA insertion generates a furin cleavage site that has been shown to increase pseudoviral infectivity, tropism, and syncytia formation in cell culture (2–5) (Fig. 1*A*). Recent structural studies have shown the presence of both prefusion (containing S1 and S2) and postfusion (containing S2 only) S structures on virions (6), indicating that the furin site is being used as virions are made in host cells. The authors suggest that the furin-cleaved S2 structures may play a role in immune system evasion, further highlighting the importance of furin cleavage and modifying factors in viral infectivity/pathogenesis.

The region proximal to the furin cleavage site contains predicted sites of *O*-glycosylation, a posttranslational modification that is catalyzed by a family of enzymes known as the UDP-GalNAc:polypeptide *N*-acetylgalactosaminyltransferases (GALNTs) (7) (Fig. 1*A*). Two of the predicted sites of *O*-glycosylation have recently been confirmed in cell culture (8). *O*-glycosylation is known to both positively and negatively influence proteolytic cleavage events in diverse proteins (9, 10), suggesting that furin cleavage of S may be modulated by host cell *O*-glycosylation. The recent emergence of highly transmissible SARS-CoV-2 variants (alpha or B.1.1.7, and delta or B.1.617.2), which contain mutations in the novel proline (P681) that lies near the sites of *O*-glycosylation and furin cleavage, has further highlighted the need to understand how changes in this region potentially influence viral infectivity (11, 12). Here we demonstrate that *O*-glycosylation decreases furin cleavage and syncytia formation in cultured cells. Moreover, we demonstrate that *O*-glycosylation in this region is dependent on P681 and that mutations in P681 result in decreased *O*-glycosylation, increased furin cleavage, and increased syncytia formation. We further define the key GALNTs responsible for glycosylating this region and examine their expression across respiratory cells that are targets for SARS-CoV-2 infection. Our results provide mechanistic insight into how P681 mutations found in recent variants of concern may contribute to increased viral infectivity by decreasing *O*-glycosylation and thus allowing increased furin cleavage. Moreover, our results further suggest that individual variation in the expression of certain GALNTs in respiratory cells (and thus the extent of S glycosylation) may influence viral transmission.

## Results

### GALNT1 Glycosylates S and Is Dependent on P681.

To investigate the role of *O*-glycosylation in modulating furin cleavage of S, we expressed GFP- and V5-labeled full-length S with or without the novel furin cleavage site (Fig. 1*B*) in a cell culture background where no *O*-glycosylation was detected and furin cleavage was active. As shown in Fig. 1*C*, S expressed in *Drosophila* S2R^+^ cells results in full-length S as well as S1 and S2 cleavage products that are the result of furin cleavage; mutation of the furin site (RRAR to AAAR; SPΔfurin-V5) abrogated cleavage, resulting in the presence of only intact S (Fig. 1*D*). Likewise, treatment with furin inhibitors decreased cleavage (*SI Appendix*, Fig. S1*A*), further demonstrating that furin cleavage is active in this cell background. S expressed in these cells displayed no reactivity to the *O*-glycan–specific lectins *Helix pomatia* agglutinin (HPA) or peanut agglutinin (PNA) (Fig. 1*E* and *SI Appendix*, Fig. S1*B*), confirming no detectable background *O*-glycosylation. Additionally, S expressed in these cells retained functional binding ability, as evidenced by binding to cells expressing the receptor hACE2 (Fig. 1 *F* and *G*). We therefore used this cell background to screen for members of the human GALNT enzyme family (7) that are capable of glycosylating S. Interestingly, only certain GALNTs were effective at glycosylating S (Fig. 1*H* and *SI Appendix*, Figs. S1*C* and S2). S glycosylation (as detected by HPA reactivity) was detected upon coexpression with GALNT1, 2, 3, 6, 10, 13, or 16 (Fig. 1*H* and *SI Appendix*, Fig. S1*C*). No glycosylation was seen upon coexpression with GALNT4, 5, 7, 8, 9, 11, 12, 14, 15, 17, 18, 19, or 20 (Fig. 1*H*).

**Fig. 2.**
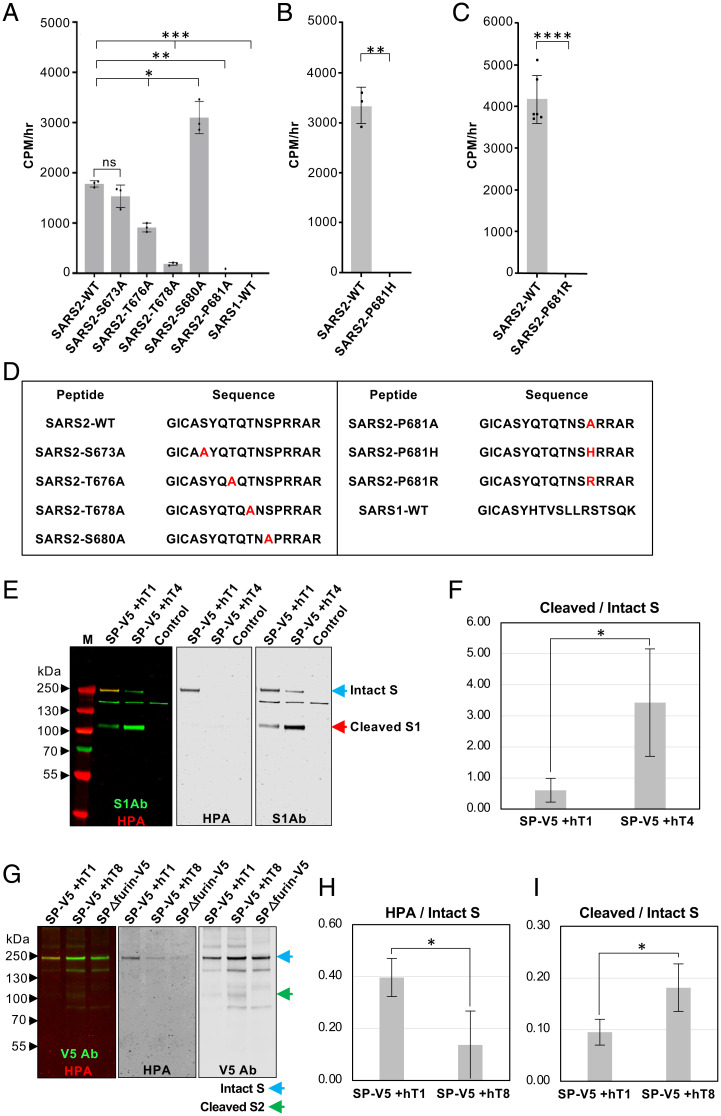
*O*-glycosylation of SARS-CoV-2 S decreases furin cleavage of S. (*A*) Enzyme assays showing GALNT1 glycosylates specific residues in the region of S proximal to the furin cleavage site and the S1/S2 border. GALNT1 glycosylates this region of SARS-CoV-2 (SARS2-WT) but not of SARS-CoV-1 (SARS1-WT). GALNT1 glycosylates T676 and T678 within the SARS-CoV-2 region. GALNT1 activity is dependent on the unique proline at position 681 (P681). (*B*) The P681H mutation found in the alpha variant abrogates GALNT1 activity. (*C*) The P681R mutation found in the delta variant abrogates GALNT1 activity. (*D*) Peptides used in enzyme assays. Mutated residues are shown in red. (*E*) Coexpression of SP-V5 with GALNT1 (hT1) (which glycosylates S) results in decreased furin cleavage relative to coexpression with GALNT4 (hT4) (which does not glycosylate S) in *Drosophila* S2R^+^ cells. The S1Ab was used to assess the ratio of cleaved to intact S (denoted by arrows). *O*-glycosylation is seen (HPA staining; red) only on the intact S that is coexpressed with GALNT1 (hT1). (*F*) Average ratios of cleaved/intact S cotransfected with either GALNT1 (hT1) or GALNT4 (hT4) from three independent experiments in *Drosophila* S2R^+^ cells. (*G*) Coexpression of SP-V5 with GALNT1 (hT1) in Vero E6 cells results in increased *O*-glycosylation of S (detected by HPA; red) and decreased furin cleavage, relative to coexpression with GALNT8 (hT8) (which does not glycosylate S). The V5 Ab was used to assess the ratio of cleaved to intact S (denoted by arrows). (*H*) S protein glycosylation (expressed as HPA/intact S protein ratio) after coexpression with either GALNT1 or GALNT8 from three independent experiments. (*I*) Average ratios of cleaved/intact S cotransfected with either GALNT1 or GALNT8 from three independent experiments. Control lanes are from cells transfected with the empty vector. M, markers. Marker size is shown in kilodaltons on the side of each panel. Error bars are SD. *P < 0.05; **P < 0.01; ***P < 0.001; ****P < 0.0001.

To assess which of the identified GALNTs are capable of glycosylating the furin proximal region of S, GALNT1, 2, 3, 6, 13, and 16 were expressed, partially purified and tested for GalNAc transfer (as described previously in ref. 13) to peptides within the furin proximal region of SARS-CoV-2 S. Enzyme assays demonstrated that only GALNT1 showed detectable transferase activity above background against this region of S (Fig. 2 *A–D* and *SI Appendix*, Fig. S3). Additionally, examining the activity of GALNT1 against peptides where each potential site of *O*-glycosylation was eliminated revealed a significant loss of activity against peptides containing mutations at T676 and T678, suggesting that these are preferred sites of GalNAc addition by GALNT1 (Fig. 2*A* and *SI Appendix*, Fig. S3*A*). T678 is one of the sites previously identified as being *O*-glycosylated in human cells by mass spectroscopy (8, 14).

We next examined the role of the novel proline at position 681 (P681) on glycosylation. Interestingly, mutation of this proline to alanine (P681A) resulted in complete abrogation of all GALNT1-mediated glycosylation (Fig. 2*A*). Similarly, mutations of this proline seen in the highly transmissible alpha (P681H) or delta (P681R) variants also resulted in a loss of GALNT1 glycosylation activity in this region (Fig. 2 *B* and *C* and *SI Appendix*, Fig. S3*B*). Finally, we compared glycosylation of this region in SARS-CoV-2 relative to the comparable region of SARS-CoV-1 (that lacks the novel PRRA insertion). As shown in Fig. 2*A*, GALNT1 was unable to glycosylate the comparable region of SARS-CoV-1. Taken together, these results suggest GALNT1 glycosylates specific residues within the furin proximal region of the SARS-CoV-2 S and that mutations at P681 abrogate glycosylation.

### *O*-Glycosylation of S Decreases Furin Cleavage.

Interestingly, our cell culture experiments revealed that *O*-glycosylation was only present on the full-length S, and never seen on either the S1 or S2 proteolytic fragments (Fig. 1*H*), suggesting that these events are mutually exclusive. To investigate whether *O*-glycosylation may interfere with furin cleavage of S, we coexpressed S with various GALNTs and assessed the ratio of cleaved to intact S. S was coexpressed with either GALNT1 (which glycosylates S) or GALNT4 (which does not glycosylate S), and ratios of S were quantitated via Western blots. As shown in Fig. 2 *E* and *F* and *SI Appendix*, Fig. S4, coexpression of S with GALNT1 resulted in *O*-glycosylation of S and a significant reduction in furin cleavage. To examine whether *O*-glycosylation modulates furin cleavage of S in mammalian cells, we performed the same experiments in Vero E6 cells, comparing the effects of GALNT1 to GALNT8 (another GALNT that does not glycosylate S) (Fig. 2 *G–I* and *SI Appendix*, Fig. S5). It is worth noting that Vero E6 cells express endogenous *Galnt1* (*SI Appendix*, Fig. S5 *G* and *H*), creating a background where S is already *O*-glycosylated to some degree (as detected by HPA reactivity) in the absence of coexpression with GALNT1 (Fig. 2*G* and *SI Appendix*, Fig. S5 *A* and *D*). As shown in Fig. 2 *G–I* and *SI Appendix*, Fig. S5 *A–F*, coexpression of S with GALNT1 resulted in increased *O*-glycosylation of intact S and decreased furin cleavage, relative to coexpression with GALNT8. Taken together, our results indicate that *O*-glycosylation of S by specific members of the GALNT family can modulate furin cleavage of S in both *Drosophila* and mammalian cells.

**Fig. 3.**
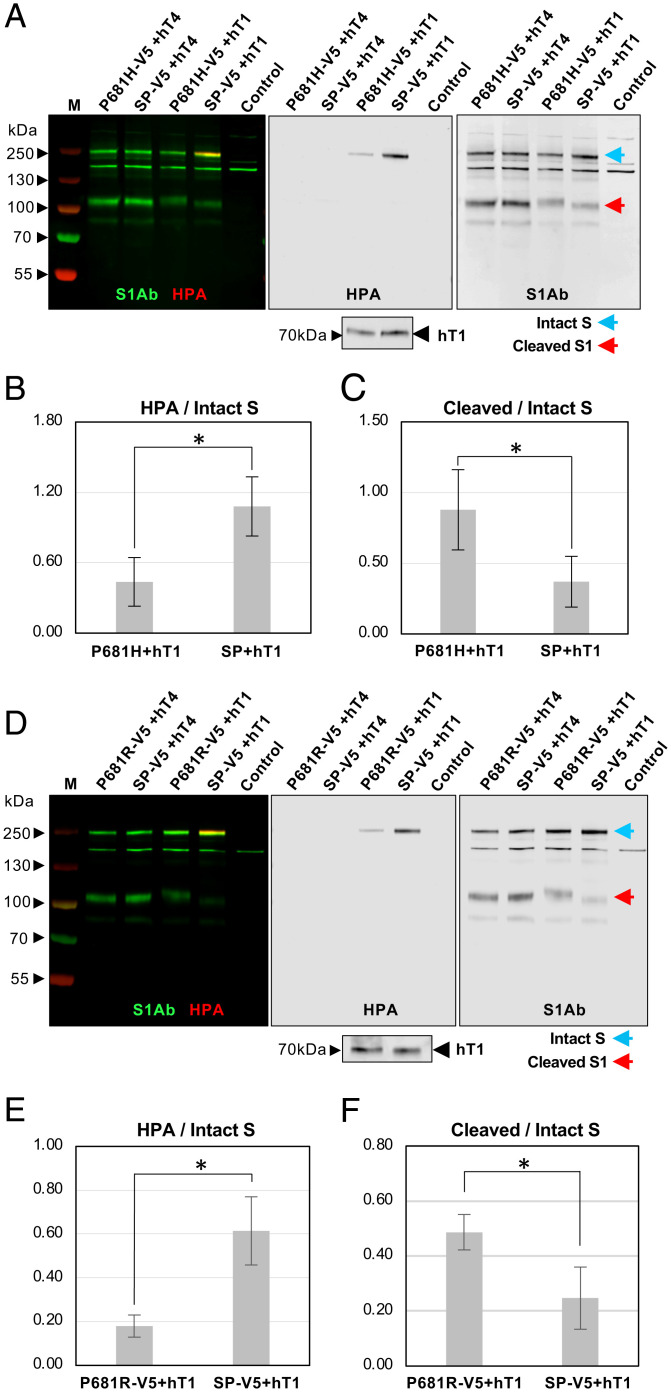
P681H and P681R decrease *O*-glycosylation and increase furin cleavage of S. (*A*) WT S (SP-V5) or S with the P681H mutation found in the alpha variant (P681H-V5) were coexpressed with GALNT4 (hT4, negative control) or GALNT1 (hT1) in *Drosophila* S2R^+^ cells and Western blotted to examine furin cleavage (detected by S1Ab; green) and *O*-glycosylation (detected by HPA; red). Full-length S is shown by the blue arrow and the cleaved S1 fragment is shown by the red arrow. *Inset* below *Center* is a duplicate blot probed with the FLAG antibody to detect levels of hT1. Control lanes are from cells transfected with the empty vector. Marker size is shown in kilodaltons on the side of each panel. The P681H mutation resulted in a significant decrease in *O*-glycosylation of S and an increase in furin cleavage of S. (*B*) S protein glycosylation (expressed as HPA/intact S protein ratio) and (*C*) cleaved/intact S from three independent experiments. (*D*) WT S (SP-V5) or S with the P681R mutation found in the delta variant (P681H-V5) were coexpressed with GALNT4 (hT4, negative control) or GALNT1 (hT1) in *Drosophila* S2R^+^ cells and Western blotted to examine furin cleavage (detected by S1Ab; green) and *O*-glycosylation (detected by HPA; red). The P681R mutation resulted in a significant decrease in *O*-glycosylation of S and an increase in furin cleavage of S. *Inset* below *Center* is a duplicate blot probed with the FLAG antibody to detect levels of hT1. (*E*) S protein glycosylation (expressed as HPA/intact S protein ratio) and (*F*) cleaved/intact S from three independent experiments. Error bars are SD. *P < 0.05.

We next assessed the role of the P681 mutations found in the alpha and delta variants on both *O*-glycosylation and furin cleavage. GALNT1 was coexpressed with either wild-type (WT) S or S containing the P681H (alpha) or P681R (delta) mutation in *Drosophila* cells (Fig. 3 and *SI Appendix*, Figs. S6 and S7). Both the P681H and the P681R mutations resulted in decreased glycosylation of S by GALNT1 and a concomitant increase in furin cleavage in cells (Fig. 3 and *SI Appendix*, Figs. S6 and S7). Interestingly, neither the P681H nor the P681R mutation showed a difference in cleavage relative to WT S in the absence of GALNT1 in these *Drosophila* cells (*SI Appendix*, Figs. S6*J* and S7*J*), indicating that furin cleavage differences between WT and P681 mutants are dependent on GALNT1-mediated *O*-glycosylation. Taken together, these results indicate that mutation of the proline at position 681 decreases *O*-glycosylation, which then results in increased furin cleavage.

### GALNT1 Decreases Syncytia Formation.

**Fig. 4.**
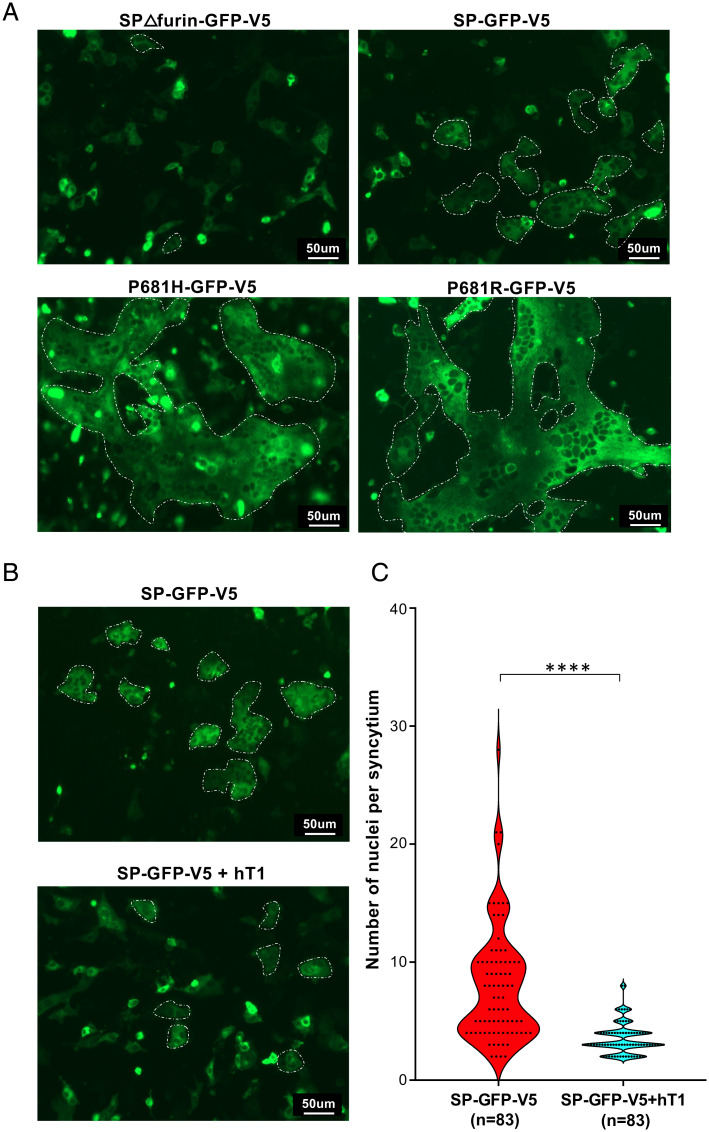
*GALNT1* expression decreases syncytia formation. (*A*) SPΔfurin-GFP-V5, SP-GFP-V5, P681H-GFP-V5, or P681R-GFP-V5 were expressed in Vero E6 cells and syncytia formation (GFP^+^ cells containing multiple nuclei) was assessed. Multinucleated regions are outlined with a white dotted line in each panel. SPΔfurin-GFP-V5 has few regions with multinucleated cells when compared to SP-GFP-V5, which has larger, more numerous multinucleated regions. P681H-GFP-V5 and P681R-GFP-V5 resulted in huge multinucleated regions, indicating that these mutations caused a dramatic increase in syncytia formation. (*B*) SP-GFP-V5 was coexpressed with or without GALNT1 (hT1) and the degree of syncytia formation was assessed. (*C*) Violin plots quantitating syncytia formation for the data shown in *B* and *SI Appendix*, Fig. S8, expressed as number of nuclei per syncytium. Each dot represents a single GFP^+^ syncytium. *n* = number of independent syncytium quantitated. ****P < 0.0001. (Scale bar, 50 μm.)

Finally, we tested the effects of P681 mutation and *O*-glycosylation on syncytia formation using Vero E6 cells. Expression of S containing the furin cleavage site has been shown previously to drive cell–cell fusion events, creating large multinucleated cells (syncytia) (5). WT S (SP-GFP-V5), S with the furin site deleted (SPΔfurin-GFP-V5), S containing the P681H mutation (P681H-GFP-V5), or S containing the P681R mutation (P681R-GFP-V5) were C-terminally tagged with GFP and expressed in Vero E6 cells. As shown in Fig. 4*A* and *SI Appendix*, Fig. S8, S lacking the furin site (SPΔfurin-GFP-V5) has few regions with multinucleated cells while SP-GFP-V5 has larger, more numerous multinucleated regions. Interestingly, both P681H-GFP-V5 and P681R-GFP-V5 resulted in very large multinucleated regions, indicating that P681 mutations increase the degree of syncytia formation relative to WT S. To assess the effect of *O*-glycosylation on syncytia formation, we next coexpressed WT S with or without GALNT1 and quantitated the number of nuclei in each syncytia. As shown in Fig. 4 *B* and *C*, coexpression of WT SP-GFP-V5 with GALNT1 resulted in a significant reduction in the degree of syncytia formed. Taken together, these data indicate that *O*-glycosylation modulates furin cleavage and syncytia formation, and mutations in P681 decrease *O*-glycosylation, resulting in increased furin cleavage and syncytia formation.

**Fig. 5.**
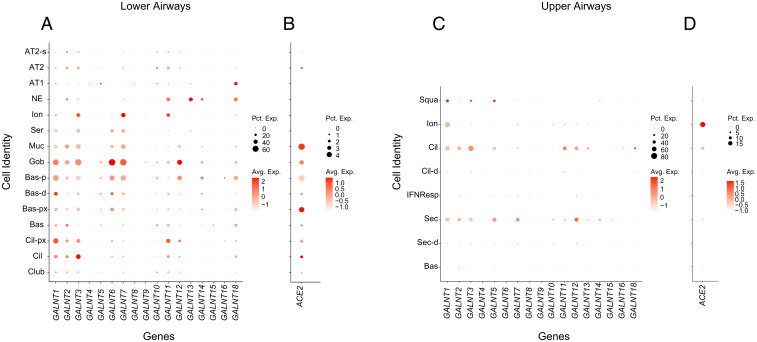
*GALNT* expression in cells of the human respiratory tract. Dot plots showing expression of *GALNT* family members (*A*) and *ACE2* (*B*) in cells of the lower respiratory tract from the cell atlas of a healthy control (15). Dot size represents the percentage of cells expressing each gene (percent expression; Pct. Exp.) and color intensity denotes degree of expression (average expression; Avg. Exp.) within each group. Cell types shown are: AT1, alveolar epithelial type-1; AT2, alveolar epithelial type-2; AT2-s, AT2-signaling; NE, neuroendocrine; Ion, ionocyte; Ser, serous; Muc, mucous; Gob, goblet; Bas-p, proliferating basal; Bas-d, differentiating basal; Bas-px, proximal basal; Bas, basal; Cil-px, proximal ciliated; Cil, ciliated; Club. Dot plots showing expression of *GALNT* family members (*C*) and *ACE2* (*D*) in cells of the upper respiratory tract from the cell atlas of a healthy control (16). Cell types shown are: Squa, squamous; Ion, ionocyte; Cil, ciliated; Cil-d, differentiating ciliated; IFNResp, IFNG responsive; Sec, secretory; Sec-d, differentiating secretory; Bas, basal.

Given the potential role of endogenously expressed host GALNTs to alter the *O*-glycosylation and therefore processing of S in cells, we next set out to define the repertoire of *GALNT*s expressed in cells of the respiratory tract likely to be infected by SARS-CoV-2. We mined the single-cell RNA-sequencing databases from cells of the lower respiratory tract of healthy controls from Travaglini et al. (15). As shown in Fig. 5, *GALNT1*, *2*, *3*, *6*, *7*, *11*, *12*, and *18* are the predominant family members expressed. In particular, abundant expression of *GALNT1* is notable across many cell types that also express ACE2 and are thus likely targets for SARS-CoV-2 infection (Fig. 5 *A* and *B*). We next examined expression levels of *GALNT*s in the upper respiratory cells of healthy controls from the dataset of Chua et al. (16) (Fig. 5 *C* and *D*). In cells expressing the highest levels of *ACE2* (ionocytes), *GALNT1* is the most abundantly expressed family member (Fig. 5 *C* and *D*).

## Discussion

Our results identify *O*-glycosylation as a modulator of furin cleavage of the S protein of SARS-CoV-2. This furin cleavage site is unique to SARS-CoV-2 and has been shown to increase pseudoviral infectivity and syncytia formation in cell culture (2–5), suggesting that it may play a role in viral transmission and disease progression in vivo. Our results demonstrate that *O*-glycosylation decreases furin cleavage of S and decreases syncytia formation in cells, raising the possibility that differences in *GALNT* expression from individual to individual could influence viral transmission in vivo. In particular, we identify one *O*-glycosyltransferase (GALNT1) expressed within ACE2^+^ cells of the upper and lower respiratory tracts, that specifically glycosylates the furin proximal region within S. GALNT1 coexpression with S in cell culture resulted in increased *O*-glycosylation and decreased furin cleavage, supporting a role for GALNT1 glycosylation in modulating SARS-CoV-2 S processing.

Interestingly, GALNT1-mediated *O*-glycosylation was dependent on P681, which is uniquely present in SARS-CoV-2. Prior biochemical and structural studies have demonstrated that certain GALNT family members have “proline pockets” within their catalytic domains and display strong preferences for sites of *O*-glycosylation that have vicinal prolines (17, 18). Indeed, mutation of this proline resulted in the loss of GALNT1 glycosyltransferase activity within the furin cleavage region in vitro. Additionally, proline mutations mimicking those seen in the highly transmissible alpha (P681H) or delta (P681R) variants showed decreased *O*-glycosylation and increased furin cleavage of S in cell culture. It is worth noting that no significant differences in furin cleavage between WT S and the P681 mutants were seen in the absence of GALNT1-mediated glycosylation, indicating that differential furin cleavage was dependent on *O*-glycosylation. Taken together, our results demonstrate that P681 mutations decrease GALNT1-mediated glycosylation, which leads to an increase in furin cleavage of S. These results provide insight into a potential functional role of P681 mutations seen in the recent variants of concern.

Finally, we demonstrate that GALNT1 coexpression with WT S can significantly reduce syncytia formation in cell culture, supporting roles for *O*-glycosylation in modulating both S-processing and S-mediated membrane fusion events. Moreover, we show that mutations in P681 resulted in a dramatic increase in syncytia formation relative to WT S, highlighting the importance of this amino acid. In summary, we demonstrate that GALNT1-mediated *O*-glycosylation, which is dependent on P681, decreases furin cleavage of S and syncytia formation in cell culture. This study provides insight into the potential functional roles of *O*-glycosylation of the SARS-CoV-2 S protein in vivo.

## Materials and Methods

*SI Appendix* provides detailed descriptions of cloning of SARS-CoV-2 S, human ***ACE2***, and ***GALNT***s; expression of spike and GALNTs in *Drosophila* S2R**^+^** cells/Vero E6 cells and Western blotting; furin inhibitor treatment in cell culture; cell imaging; cell syncytia formation assay; purification of human GALNT1; and quantitative real-time PCR.

### Enzyme Assays.

Expression of recombinant GALNTs was performed either using *Pichia pastoris* or COS7 cells as described previously (13) and as described in *SI Appendix*.

### scRNA-Sequencing Analysis.

Details are described in *SI Appendix*. The datasets are available for download from their respective published sources (15, 16).

## Supplementary Material

Supplementary File

## Data Availability

All study data are included in the article and/or supporting information.
