# Supplementary material for: Furin cleavage of the SARS-CoV-2 spike is modulated by O-glycosylation
Source: Proc Natl Acad Sci U S A. 2021 Nov 3;118(47):e2109905118. doi: 10.1073/pnas.2109905118 (PMC8617502; doi:10.1073/pnas.2109905118)
Supplement: Supplementary File [file pnas.2109905118.sapp.pdf]

PNAS
www.pnas.org

Supplementary Information for

FURIN CLEAVAGE OF THE SARS-COV-2 SPIKE IS MODULATED BY O-GLYCOSYLATION

Liping Zhang, Matthew Mann, Zulfeqhar A. Syed, Hayley M. Reynolds, E Tian, Nadine L. Samara, Darryl C. Zeldin, Lawrence A. Tabak, and Kelly G. Ten Hagen

Corresponding author: Kelly G. Ten Hagen
Email: Kelly.Tenhagen@nih.gov

This PDF file includes:

Supplementary Materials and Methods
Figures S1 to S8 and legends
Table S1

Supplementary Materials and Methods

Cloning of SARS-CoV-2 *Spike* (*S*), human *ACE2* and *GALNT*s. The codon optimized cDNA of full-length Spike (Genscript) was amplified by PCR and digested by HindIII and NotI, then cloned into pIB/V5-His vector (Invitrogen), fused with a V5 or GFP-V5 epitope tag at the C-terminus. To make mutations to remove the furin cleavage site (spikeΔfurin), two arginines at position 682 and 683 were changed to alanines using the QuickChange II XL site-directed mutagenesis kit (Agilent Technologies) and the plasmid pIB-spike-V5 was used as a template. Mutagenic primers were designed using the QuickChange Primer Design Program available online. The sense primer sequence used was 5'-cgagcacggcgagctgagttagtctggg-3' and anti-sense primer sequence was 5'-cccagactaactcagctcgccgtgctcg-3'. To make the P681H mutation, the sense primer used was 5'-agcgagcacggcgatgtgagttagtctgg-3' and anti-sense primer was 5'-ccagactaactcacatcgccgtgctcgct-3'. To make the P681R mutation, the sense primer used was 5'-gcgagcacggcgacgtgagttagtctg-3' and anti-sense was 5'-cagactaactcacgtcgccgtgctcgc-3'. Reactions and transformation were performed according to the manufacturer's instructions. DNA sequencing was performed to verify the mutations. The cDNA of *hACE2* (Genscript) was amplified by PCR and digested by KpnI and NotI, then cloned into pIB/V5-His vector, fused with mAPPLE/V5 epitope at the C-terminus. The cDNAs of *GALNTs* 1-20 (Genscript) were amplified by PCR and digested by BamHI and NotI (*GALNT1, 2, 3, 4, 9, 13, 14, 17, 18, 19, 20*) or HindIII and NotI (*GALNT5, 6, 8, 10, 11, 15, 16*) or HindIII and XhoI (*GALNT12*), or SacI and NotI (*GALNT7*), then cloned into the pIB vector, fused with FLAG epitope tag at the C-terminus. pcDNA3.1-spike and pcDNA-3.1- spikeΔfurin (Genscript) were fused with V5 or GFP-V5 epitope tag at the C-terminus to make

pcDNA3.1-spike-V5, pcDNA3.1-spike-GFP-V5, pcDNA-3.1-spikeΔfurin-V5 and pcDNA-3.1-spikeΔfurin-GFP-V5. To make the P681H and P681R mutations, mutagenic primers were designed using the QuickChange Primer Design Program available online. For the P681H mutation, the sense primer sequence used was 5'-ctccgggctcttctgtgagagtttgtctggg -3' and anti-sense primer sequence was 5'-cccagacaaactctcacagaagagcccggag-3'. For the P681R mutation, the sense primer sequence used was 5'-tccgggctcttctgcgagagtttgtctgg-3' and anti-sense primer sequence was 5'-ccagacaaactctcgcagaagagcccgga-3'. Plasmid pcDNA3.1-spike-V5 or pcDNA3.1-spike-GFP was used as a template.

Expression of Spike and GALNTs in *Drosophila* S2R+ cells/Vero E6 cells and western blotting. The plasmids pIB-spike-V5 with or without pIB-GALNTs-FLAG were transfected to S2R+ cells (DGRC) using Effectene transfection reagent (Qiagen) according to manufacturer's instructions. For expression of Spike in Vero E6 cells (ATCC), the cells were transfected with pcDNA3.1-spike-V5 with pcDNA3.1-GALNT1-FLAG or pcDNA3.1-GALNT8-FLAG (pcDNA3.1-GALNTs-FLAG vectors were purchased from Genscript) using Lipofectamine 3000 reagent (Invitrogen) according to manufacturer's instructions.

For western blotting, cells were lysed with RIPA buffer (Sigma) containing 1X Halt Protease Inhibitor (Thermo Scientific) 3-4 days after transfection. Protein extracts were incubated with agarose-immobolized V5 antibody (Bethyl) overnight at 4°C. Then beads were collected and washed with PBS four times and protein samples were mixed with LDS sample buffer (Invitrogen) containing BME. Proteins were analyzed by NuPAGE 4–12% Bis-Tris gels and transferred onto nitrocellulose membranes. The membranes were blocked

with Odyssey Blocking Buffer (PBS-based) (Li-COR), then incubated with anti-V5 antibody (dilution 1:1000, Invitrogen) or anti-S1 antibody (dilution 1:1000, GeneTex) overnight at 4 °C. After washing with PBS containing 0.1% Tween-20 (PBST), the membrane was incubated with IRDye 680LT-conjugated anti-mouse IgG (1:5000, Li-COR) or 680LT-conjugated anti-rabbit IgG (1:5000, Li-COR) and IRDye 800CW (Li-COR) labeled HPA (Helix pomatia lectin, Sigma) or PNA (peanut agglutinin, Sigma) for 1hr at room temperature. After washing with PBST and PBS, the membranes were scanned by a Li-COR Odyssey Infrared Imaging System. The protein band intensity was measured using ImageJ to calculate the ratio of cleaved protein/ intact protein and the ratio of HPA/ intact protein.

Furin inhibitor treatment in cell culture. 24 hrs after transfection, the furin inhibitor decanoyl-RVKR-CMK (Tocris) was added to the medium at final concentrations of 20uM, 50uM or 100uM. Cells were collected 72 hrs after transfection and lysed with RIPA buffer (Sigma) containing 1X Halt Protease Inhibitor (Thermo Scientific). The cell lysates were used for immunoprecipitation and western blotting experiments.

Cell imaging. S2R+ cells were seeded into 24-well plates. One well of cells was transfected with pIB-spike-GFP plasmid and another well of cells was transfected with pIB-hACE2-mAPPLE. 24 hrs after transfection, two wells of cells were suspended by gently pipetting and mixing. The mixed cells were transferred to glass bottom dish (MatTek) and incubated at 25°C for 24 hrs. After incubation, cells were fixed with 4% PFA, washed with PBST and mounted with VECTASHIELD Mounting Medium with DAPI (Vector Laboratories). The cells were imaged on Nikon A1R+ confocal microscope. Images were processed using Fiji.

Cell syncytia formation assay. Vero-E6 cells were seeded into 24-well plates. The cells were transfected with pcDNA3.1-spike-GFP-V5; pcDNA-3.1-spikeΔfurin-GFP-V5; pcDNA-3.1-P681H-GFP-V5; pcDNA-3.1-P681R-GFP-V5 or co-transfected with pcDNA3.1-spike-GFP-V5 and pcDNA3.1-GALNT1-FLAG-V5. 48 hrs after transfection, cells were imaged on an EVOS microscope to check for syncytium formation. Images were processed using ImageJ. The number of nuclei in each syncytium were counted for cells transfected with pcDNA3.1-spike-GFP or co-transfected with pcDNA3.1-spike-GFP plus pcDNA3.1-GALNT1-FLAG. 83 syncytia were counted for each sample. The data were analyzed (two-way ANOVA) and graphed using Prism 9.

Purification of human GALNT1. Human (h) GALNT1 (residues 44-559) was cloned into the vector pKN55 containing an N-terminal His_6-TEV tag for expression in *Pichia pastoris*. The vector was linearized with PmeI and transformed into *P. pastoris* SMD1168 cells (Invitrogen) by electroporation. For large-scale expression of hGALNT1, 2 L of cells were grown to an OD600 ~ 20 at 29 °C in BMGY media. The cells were pelleted by centrifugation at 1500 X g for 10 min. To induce expression of secreted protein, the cells were resuspended in ¼ volume of BMMY media and incubated at 20 °C for 24 h while shaking. The supernatant was collected by centrifugation at 1500 X g for 10 min, filtered, and followed by the addition of 50 mM Tris pH 7.5 and 10 mM β-mercaptoethanol. The following purification steps were conducted at 4 °C. First, supernatant was loaded onto a 5 ml HisTrap HP column (GE Healthcare) pre-equilibrated with 25 mM Tris, 250 mM NaCl, 10 mM βME, pH 7.5. The protein was then eluted using a gradient of 0-500 mM imidazole over 10 CV. To cleave the His_6-Tag, hGALNT1 fractions were incubated with ~1 mg of His_6-TEV overnight at 4 °C while dialyzing into 500 ml of equilibration buffer

containing 25 mM imidazole. Untagged hGALNT1 was further purified by loading the sample onto a 1 ml HisTrap HP column (GE Healthcare) followed by washing with 6 ml of dialysis buffer. The flow-through and wash were pooled, and 30 % glycerol was added to hGALNT1 while stirring. The purified hGALNT1 was aliquoted and frozen in LN_2 prior to storage at -80 °C. The yield was 1 mg/L. Stock concentration of hT1 used in assays is ~1.3 μM. Final concentration in 25 μl reaction is 0.052 μM.

Enzyme assays. Expression of recombinant GALNTs was performed either using *Pichia pastoris* or COS7 cells as described previously and referenced in the main text, as well as described herein. Peptide substrates were synthesized by Peptide 2.0. Peptide stocks were prepared by resuspending in water to 12.5 mM, aliquoted (5-10 μl), and stored at -30°C with the exception of SARS2-P681R, which was initially resuspended in 20 % acetic acid to a concentration of 9.8 mM and then diluted to 1.8 mM with buffer containing 40 mM cacodylate pH 6.8, 40 mM βME, and 0.7% NaOH to adjust the pH to ~7. Assays were performed as described previously and referenced in the main text. Reactions were run for 30 min at 37°C in 25 μl final volumes consisting of the following: 0.5 mM acceptor substrate, 7.3 μM UDP-[^{14}C]-GalNAc (54.7 mCi/mmol; 0.02mCi/ml), 44 μM cold UDP-GalNAc, 10 mM $MnCl_2$, 40 mM cacodylate (pH 6.5), 40 mM 2-mercaptoethanol and 0.1 % Triton X-100. Reactions were then quenched with 30 mM EDTA. Glycosylated products were separated from unincorporated UDP-[^{14}C]-GalNAc by anion exchange chromatography using AG1-X8 resin columns (Bio-Rad #1401454), and product incorporation was determined by liquid scintillation counting (Beckman Coulter LS6500). Reactions without acceptor peptide were also used to generate background values that were subtracted from each experimental value. Assays for each peptide substrate were run in

duplicate or triplicate and repeated three times. Experimental values for each substrate were then averaged and standard deviations were calculated. Enzyme activity (initial rate) is expressed as CPM/hr.

Quantitative real-time PCR. DNase-free RNA was isolated by using PureLink RNA Mini Isolation Kit (Ambion). cDNA synthesis was performed by using the iScript cDNA Synthesis Kit (Bio-Rad). Human PCR primers (Table S1) were designed by using Beacon Designer software (Bio-Rad). Quantitative PCR was performed on a CFX96 real-time PCR thermocycler (Bio-Rad) using the SYBR-Green PCR Master Mix (Bio-Rad). Quantitative PCR was performed in triplicate. Gene expression levels were normalized to human *29S* rRNA and plotted as gene expression level and relative expression levels to a housekeeping gene. Values represent mean value.

scRNASeq Analysis. Published datasets for upper and lower airways were retrieved as R object data format (.rds file) and the encapsulated Seurat (v.3.2.3) object was updated to Seurat v3 object using the Seurat UpdateSeuratObject() function. We subset the data for the Epithelial cell compartment from the upper and lower airways atlas and the expression of *GALNTs* and *ACE2* is illustrated as dot plots using the R ggplot2 (v.3.3.2) library. Individual dot plots for upper and lower airways were assembled in Adobe Illustrator (v.24.3) to generate a final figure. The datasets are available for download from their respective published sources referenced in the main text.

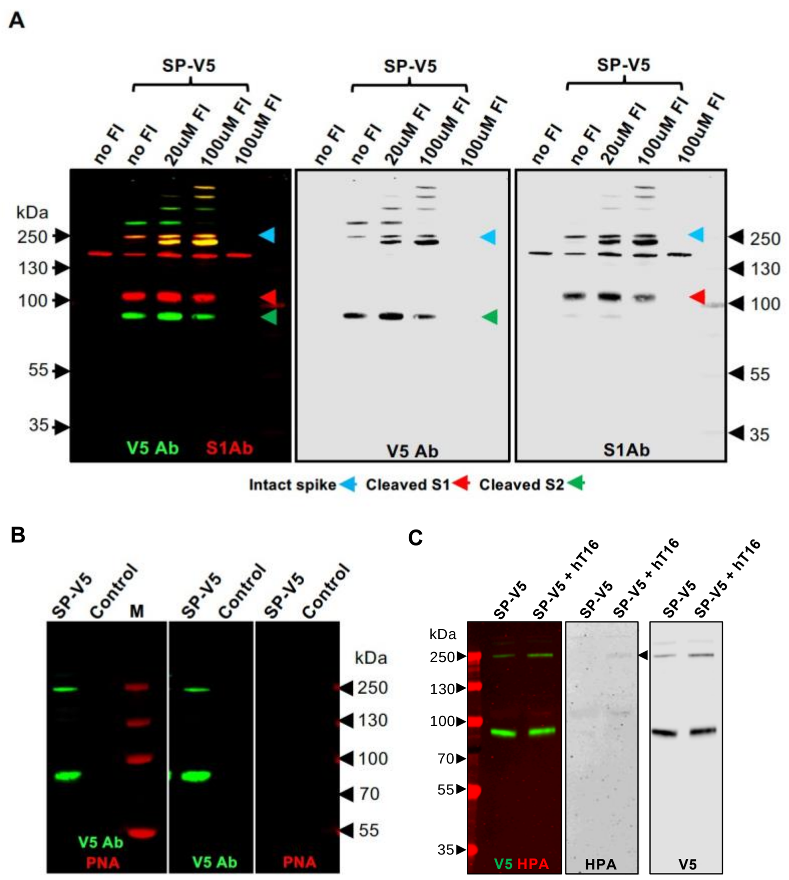

Fig. S1. **Furin cleavage of S expressed in *Drosophila* S2R+ cells.** (A) Decreased cleavage of S expressed in *Drosophila* S2R+ cells is seen upon incubation with increased concentrations of a furin inhibitor (FI). V5 Ab was used to detect intact S (blue arrow) and the cleaved S2 fragment (green arrow). The S1 Ab was used to detect the cleaved S1 fragment (red arrow). First and last lanes of each gel contains the empty vector. (B). S expressed in *Drosophila* S2R+ cells (as detected by V5 Ab) is not PNA (red) reactive, indicating no detectable PNA-reactive O-glycans. Control represents cells transfected with the empty vector. (C) Replicate western demonstrating O-glycosylation of WT SP-V5 by GALNT16. O-glycosylation was assessed by staining with the O-glycan-specific lectin HPA (red) and S was detected via staining with the V5 Ab (green). M, markers. Marker size is shown in kDa on the side of each panel.

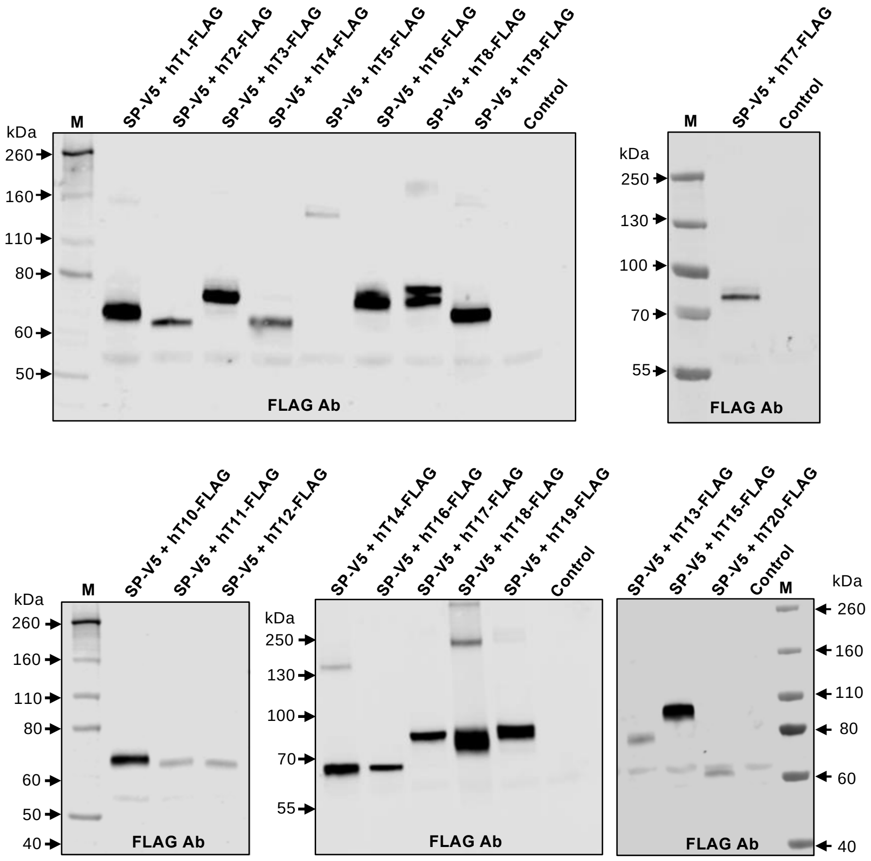

Fig. S2. Expression of GALNTs in *Drosophila* S2R+ cells. Protein extracts from cells expressing SP-V5 along with individual members of the human GALNT family that were FLAG tagged were western blotted using the FLAG antibody to verify GALNT expression. Control represents cells transfected with the empty vector. M, markers. Marker size is shown in kDa on the side of each panel.

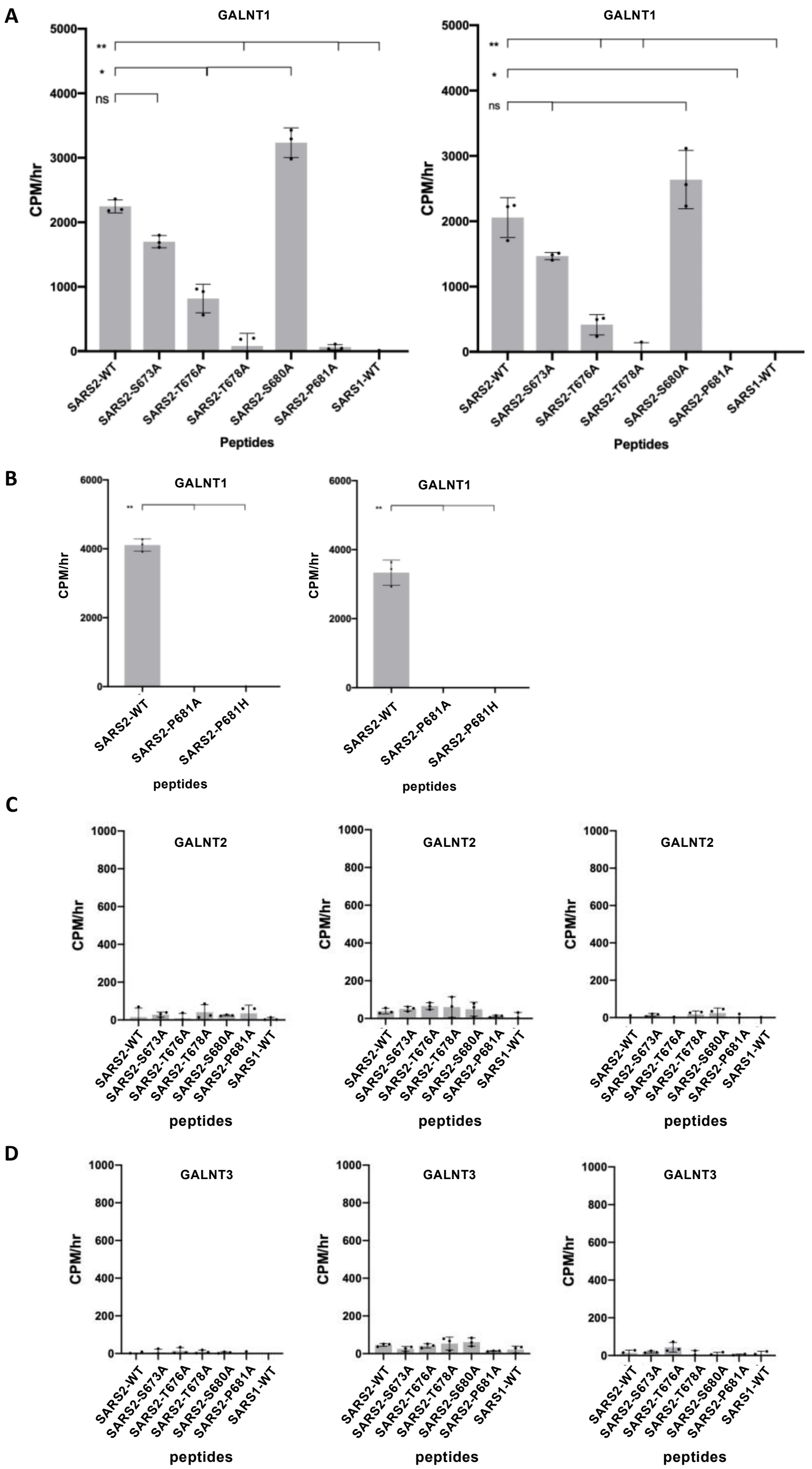
A
GALNT1
CPM/hr
Peptides
SARS2-WT
SARS2-S673A
SARS2-T676A
SARS2-T678A
SARS2-S680A
SARS2-P681A
SARS1-WT
**
*
ns
B
GALNT1
CPM/hr
peptides
SARS2-WT
SARS2-P681A
SARS2-P681H
**
C
GALNT2
CPM/hr
peptides
D
GALNT3
CPM/hr
peptides

E

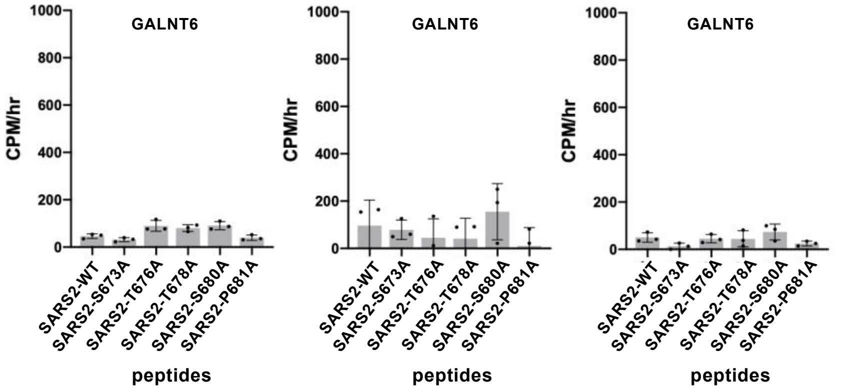

F

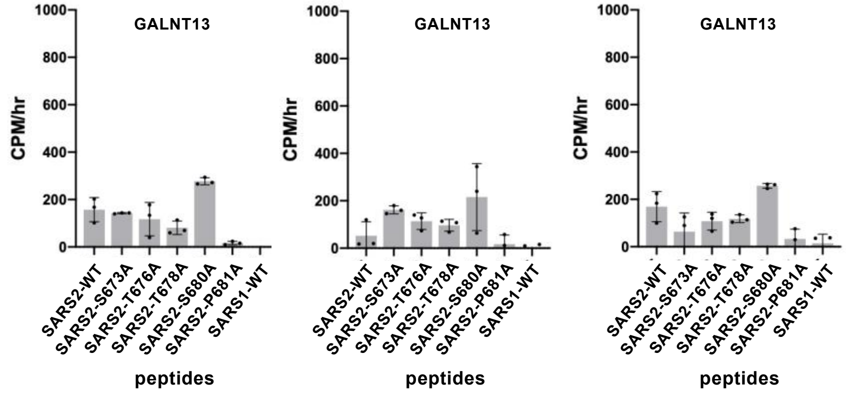

G

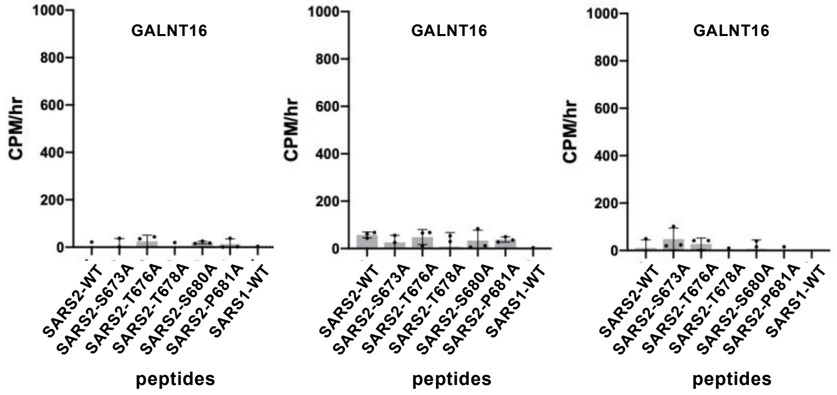

Fig. S3. O-glycosylation of SARS-CoV-2 S by GALNT1. (**A**) Replicates of enzyme assays testing for O-glycosylation of peptides from the furin proximal region of the SARS-CoV-2 S and the related region of SARS-CoV-1 S using GALNT1. (**B**) Replicates of enzyme assays testing GALNT1 activity on SARS-CoV-2 peptides containing the mutation found in the Alpha variant (P681H). GALNT1 activity is dependent on the unique proline at position 681 (P681). Replicates of enzyme assays testing activity against the SARS-CoV-2 peptides using GALNT2 (**C**), GALNT3 (**D**), GALNT6 (**E**), GALNT13 (**F**), and GALNT16 (**G**). (**B**) Peptide sequences are as shown in the main text. Each data point represents an individual assay. Error bars are SD. ns=not significant. **P*<0.05;***P*<0.01.

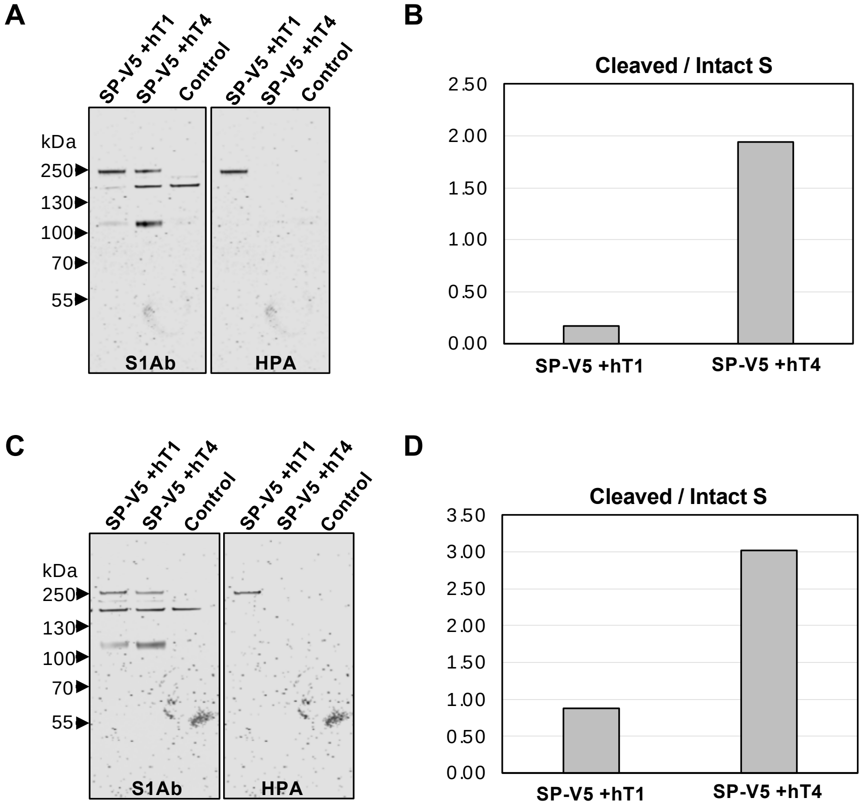

Fig. S4. O-glycosylation of SARS-CoV-2 S decreases furin cleavage in *Drosophila* S2R+ cells. Independent replicates of experiments coexpressing S with GALNT1 (hT1) (which glycosylates S) or GALNT4 (hT4) (which does not glycosylate S) in *Drosophila* S2R+ cells. S1Ab was used to detect cleaved and intact S via western blots (**A** and **C**). O-glycosylation is seen (HPA staining) only on the intact S coexpressed with GALNT1. Ratios of cleaved to intact S were quantitated (**B** and **D**). Control represents cells transfected with the empty vector. Size markers (kDa) are shown to the left of each blot.

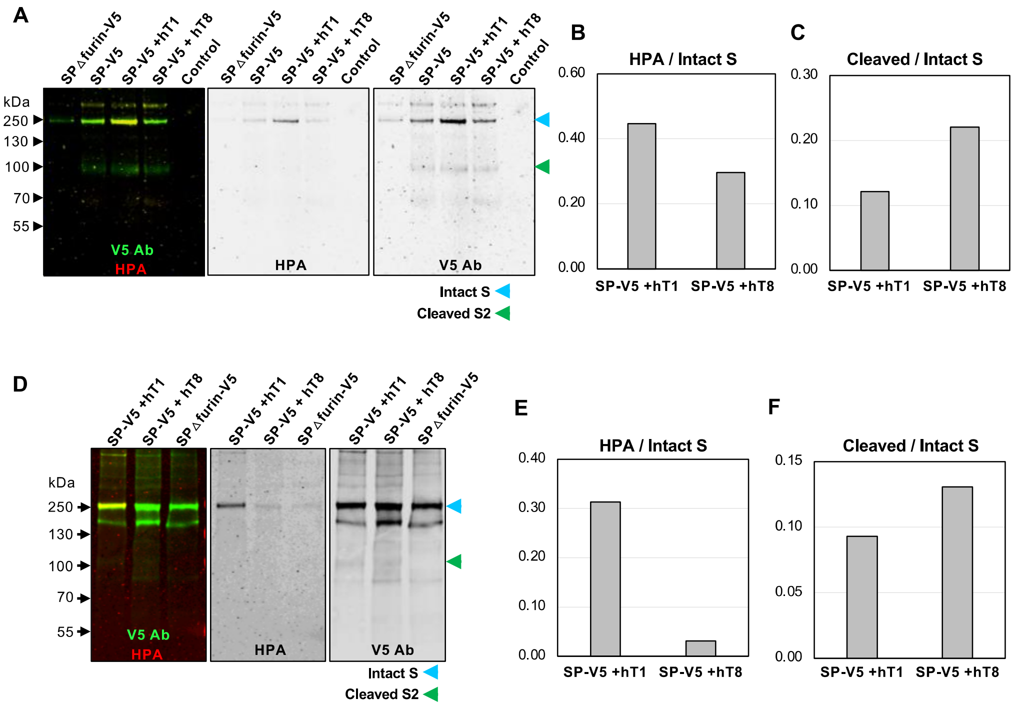

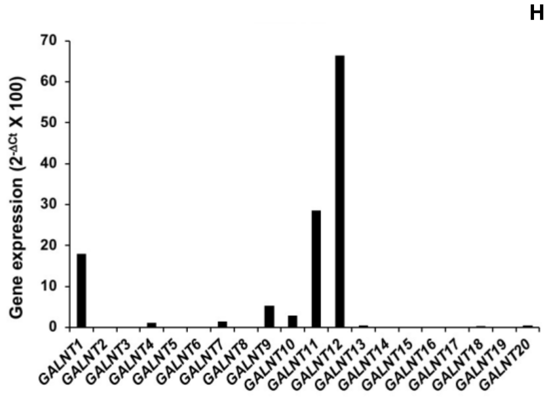

H

Gene	CT	SD
hS29	19.14	0.12
GALNT1	24.94	0.07
GALNT2	ND	NA
GALNT3	33.64	0.52
GALNT4	29.02	0.20
GALNT5	36.36	0.62
GALNT6	36.22	1.09
GALNT7	28.58	0.07
GALNT8	ND	NA
GALNT9	26.69	0.43
GALNT10	27.60	0.08
GALNT11	24.27	0.03
GALNT12	23.05	0.04
GALNT13	30.34	0.44
GALNT14	ND	NA
GALNT15	ND	NA
GALNT16	33.35	0.70
GALNT17	34.12	NA
GALNT18	30.80	0.16
GALNT19	37.65	0.78
GALNT20	30.28	0.30

Fig. S5. O-glycosylation of SARS-CoV-2 S decreases furin cleavage in Vero E6 cells. Independent replicates of experiments coexpressing S with GALNT1 (hT1) (which glycosylates S) or GALNT8 (hT8) (which does not glycosylate S) in Vero E6 cells. V5 Ab was used to detect cleaved and intact S via western blots (**A** and **D**). O-glycosylation (HPA staining) is increased on intact S coexpressed with GALNT1 relative to GALNT8 (**B** and **E**). Ratios of cleaved to intact S from each experiment were quantitated (**C** and **F**). Size markers (kDa) are shown to the left of each blot. Expression of *GALNT* family members was assessed by QPCR in Vero E6 cells (**G**). Cycle threshold (CT) value and standard deviation (SD) of *h29S* and *GALNT* genes in Vero E6 cells (**H**) are shown. ND, not detected. NA, not available.

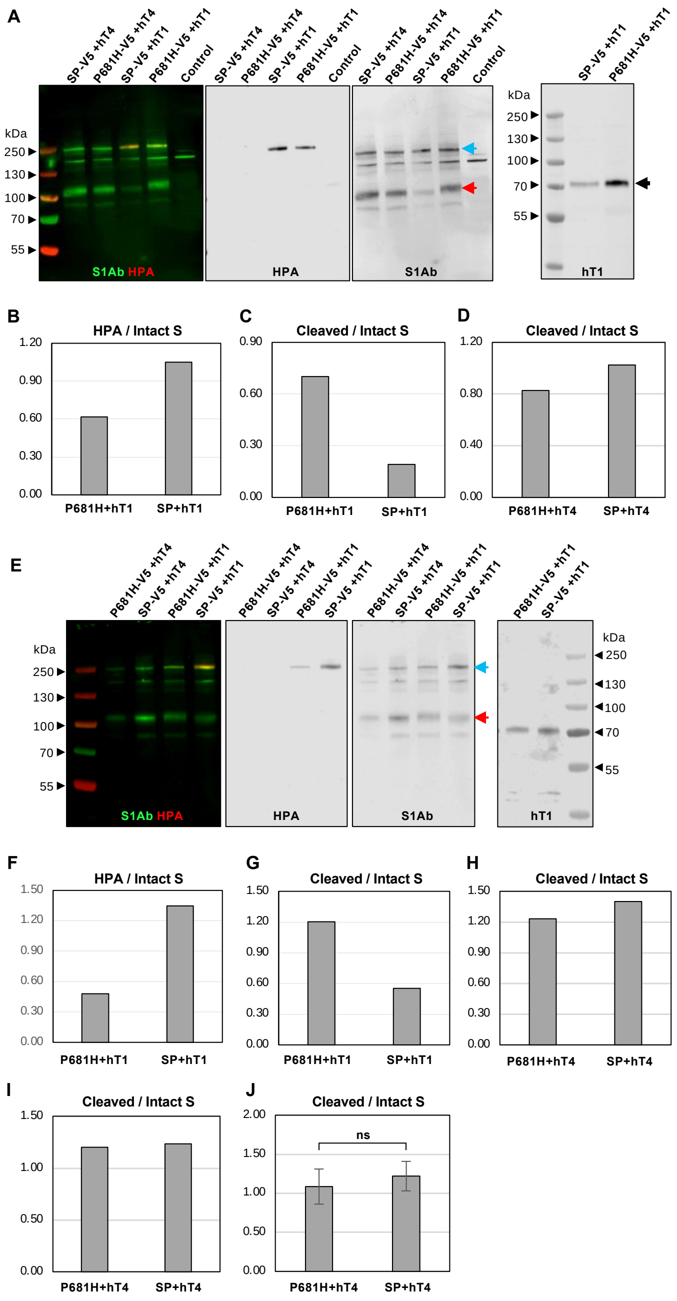

Fig. S6. P681H decreases O-glycosylation and increases furin cleavage. Independent replicates of experiments coexpressing WT S (SP-V5) or S containing the P681H mutation (P681H-V5) with either GALNT4 (hT4) or GALNT1 (hT1) in *Drosophila* S2R+ cells. The S1 Ab (green) was used to detect cleaved and intact S, HPA (red) was used to detect O-glycosylation and anti-FLAG was used to detect levels of GALNT1 via western blots (**A** and **E**). Ratios of O-glycosylation were quantitated (**B** and **F**). Ratios of cleaved to intact S from each experiment were quantitated (**C, D, G and H**). (**I**) Cleavage ratios of S from experiment shown in Fig. 3A. (**J**) Average cleavage ratio from 3 independent experiments for P681H-V5 coexpressed with GALNT4 versus SP-V5 coexpressed with GALNT4, indicating that no difference in furin cleavage between WT S and the P681H mutant is seen in the absence of O-glycosylation. Size markers (kDa) are shown to the left and right of each blot.

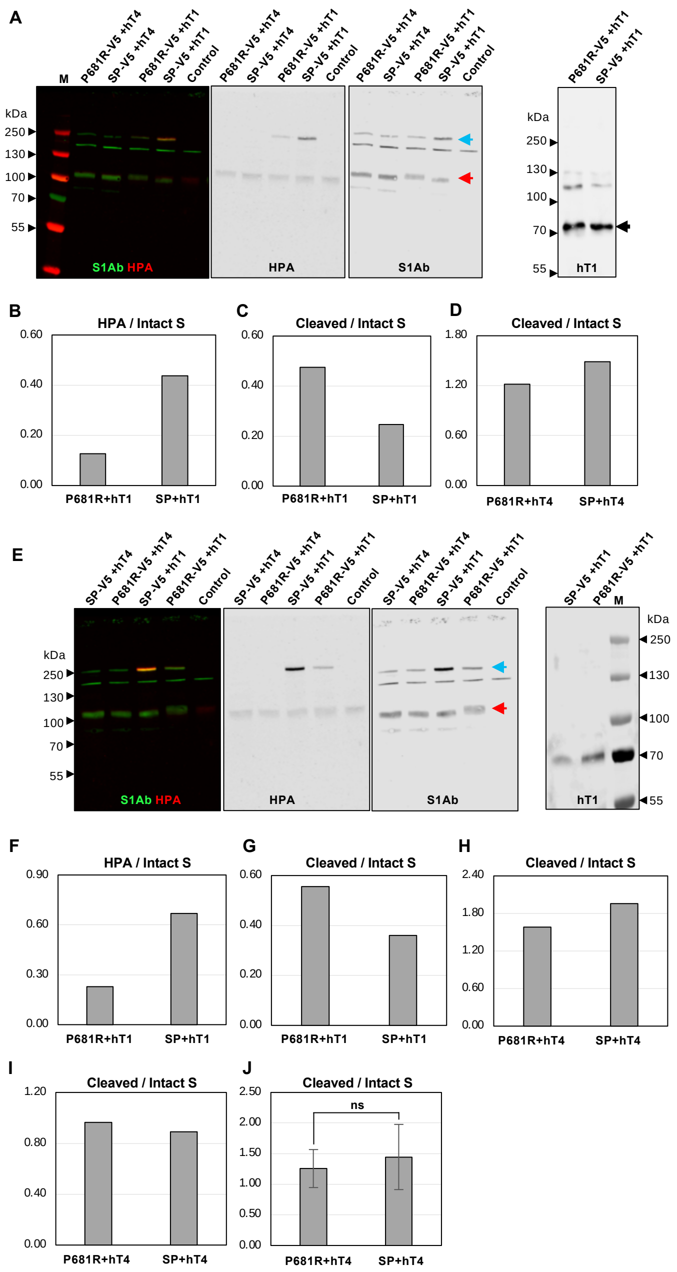

Fig. S7. P681R decreases O-glycosylation and increases furin cleavage. Independent replicates of experiments coexpressing WT S (SP-V5) or S containing the P681R mutation (P681R-V5) with either GALNT4 (hT4) or GALNT1 (hT1) in *Drosophila* S2R+ cells. The S1 Ab (green) was used to detect cleaved and intact S, HPA (red) was used to detect O-glycosylation and anti-FLAG was used to detect levels of GALNT1 via western blots (**A** and **E**). Ratios of O-glycosylation were quantitated (**B** and **F**). Ratios of cleaved to intact S from each experiment were quantitated (**C, D, G and H**). (**I**) Cleavage ratios of S from experiment shown in Fig. 3D. (**J**) Average cleavage ratio from 3 independent experiments for P681R-V5 coexpressed with GALNT4 versus SP-V5 coexpressed with GALNT4, indicating that no difference in furin cleavage between WT S and the P681R mutant is seen in the absence of O-glycosylation. Size markers (kDa) are shown to the left and right of each blot.

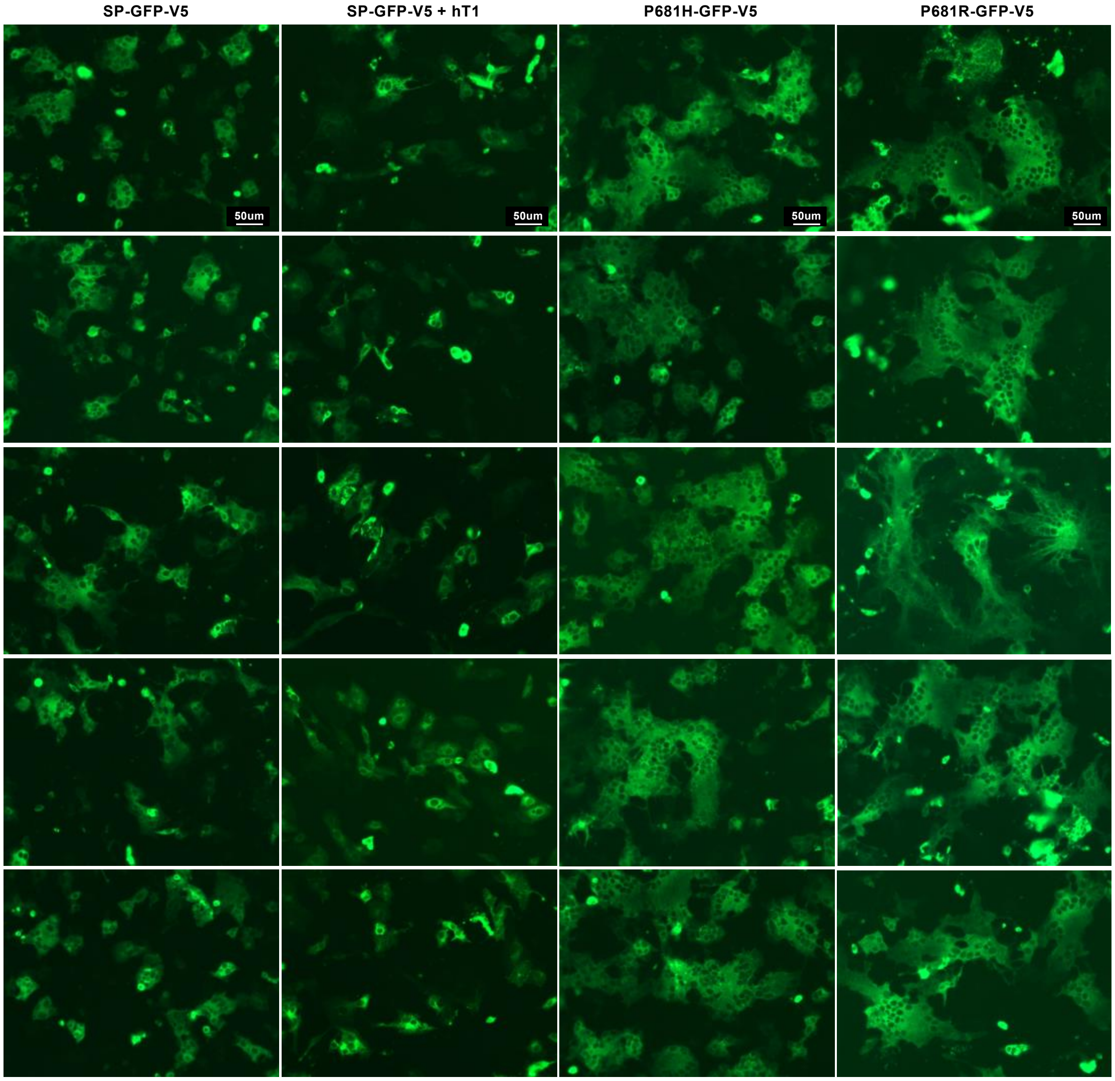

Fig. S8. ***GALNT1*** **expression decreases syncytia formation.** SP-GFP-V5 coexpressed with or without GALNT1 (hT1); P681H-GFP-V5; or P681R-GFP-V5 were expressed in Vero E6 cells and syncytia were visualized as GFP+ cells containing multiple nuclei. Multiple fields of view for each transfection are shown. Scale bar=50µm.

Table S1. PCR primer sequences used for QPCR

Gene	Sense primer sequence	Anti-sense primer sequence
GALNT1	GCAGGCAGTGCTATTTCTTGTG	CTGCTTCCTTTCAAATTTCCTGGG
GALNT2	CCTCTCAGCCTTCCTATCATCC	CGCAACCGCAGTAAGTCACT
GALNT3	CTCGGTTCTGCCTCTCCA	ACAGTTGCGGCTCAGTAGA
GALNT4	GCGGTGAGGTGGACTTGG	GGAGGTCTGAGAGCCTTCTTGA
GALNT5	CCACGCAGGCAGAGACTGACAA	CAGCAGCAGCAGCAGCAGAG
GALNT6	GGGAGGGTTCAGGGCAGCAATG	GGGAGGGTGGGCGTAGAGATGG
GALNT7	GGCTAGTGGTCCTCTGGTCTTCC	CTCTGTCTTCCCTCATCCTGCTCA
GALNT8	CTCCAGACCTGTAGCACGCAAGT	TGATGACCCAGATCCAGCACCAT
GALNT9	GGCAGACACAGCAGGACA	TGTTTGAGTTGGCATCACTGTC
GALNT10	AAGGAGCCAGCCAGGTGTAATAC	CAGTTGTGATGATGGTGGGAGGTT
GALNT11	GCCACGGGTCAGGAGGAT	TGAAGAGGAGCCATCGCAGAT
GALNT12	CAGGCTGCGAGGAAGGAGTC	GGTGGGTTCTGGTCACTGCTTAG
GALNT13	CGTCCACACCACTCACCACACAT	TGGGGCAGAGGGAACAAAACACT
GALNT14	TCGTCGTCAACCCATGTGAGTCCT	GCTGCCCAGTTTCCAGTCTGTTCT
GALNT15	AACACTGGACTTGGGCTCTG	CACCTGCTCCTGCCTGAC
GALNT16	CCTGAACCTCTGCTCTGGATTG	TGCGGACCACAGACACTTG
GALNT17	AACAGCGGAGCGTGAGAG	GGAGTCCAGCCAGGAAGTG
GALNT18	CACCGTGGATGATGATGACAAC	AACTCCAGGTCGCTATTCTCC
GALNT19	AGACGCCTTCCACGAGAT	CACGATGTCCTCCAGCAG
GALNT20	CCTCCTGTGTCTCATCTCTTGC	TGGTCCTCACTGCCTCCTT
